# Gain or loss? The well-being of women in self-employment

**DOI:** 10.3389/fpsyg.2022.986288

**Published:** 2022-09-16

**Authors:** Lin Xiu, Yufei Ren

**Affiliations:** ^1^Department of Management Studies, University of Minnesota Duluth, Duluth, MN, United States; ^2^Department of Economics and Health Care Management, University of Minnesota Duluth, Duluth, MN, United States

**Keywords:** self-employment, well-being, entrepreneurship, gender, gender equality

## Abstract

Using data from the Chinese Household Income Project survey, we find that self-employed women have lower levels of well-being compared with their male counterparts. When comparing individuals' well-being in self-employment and wage-employment, we discover that self-employed men have higher levels of health, the standard of living, satisfaction, and life satisfaction compared with wage-employed men, whereas self-employed women have lower levels of health and life satisfaction than their counterparts in wage-employment. Furthermore, if a given self-employed man or woman had been selected for wage employment, their well-being would not improve (controlling for individual characteristics that affect the likelihood to enter self-employment). Hence, self-employed women face a double challenge: lower well-being than both self-employed men and wage-employed women. The article discusses recommendations for future research and policy implications.

## Introduction

Recent studies have examined gender differences in entrepreneurship, such as the income return to an entrepreneurial career (Xiu and Gunderson, 2021), financial challenges women encounter when pursuing entrepreneurship (Gupta and Mirchandani, 2018), and institutional and network barriers that women entrepreneurs might face (Zhao and Yang, 2021). However, despite recent research on the link between entrepreneurship and well-being (Wiklund et al., 2019), little is known about gender differences in entrepreneurs' well-being compared with those in wage employment. This study addresses the following questions: is there a gender difference between the well-being of male and female entrepreneurs? Do self-employed women and men have higher or lower levels of well-being than the wage-employed? Is there a gender difference in the expected well-being gain or loss if a given self-employed woman or man had been in a different type of employment?

The current literature on entrepreneurship and well-being shows mixed results. Some studies reveal that entrepreneurship leads to better well-being outcomes because it is associated with greater autonomy, self-actualization, and flexibility (Nikolova, 2019), whereas others find that self-employment could lead to lower well-being due to long working hours and stress (Baron et al., 2016). Establishing the relationship between self-employment and well-being is challenging because two mechanisms occur simultaneously: a selection effect (i.e., healthier or less healthy individuals may choose to join self-employment) and a contextual effect (i.e., the effect of self-employment on well-being). Several recent studies have attempted to account for this issue. For instance, using longitudinal data in the United Studies, Rietveld et al. (2015) show that cross-sectional differences in health between the self-employed and wage-employed are mainly due to the selection of comparatively healthier individuals into self-employment. Using the Korean Longitudinal Study of Aging, Ahn (2020) shows that self-employment has a negative effect on general and mental health and a positive effect on cognitive functioning. Using data collected in Germany, Nikolova (2019) finds that entrepreneurship leads to better health outcomes for those who transit from regular employment to self-employment. This recent evidence sheds important light on the relationship between self-employment and well-being.

An area that remains less studied is the heterogeneity of well-being outcomes for self-employed men and women. Well-being is dependent on social status, family, and employment circumstances (Abreu et al., 2019), which tend to be drastically different for men and women. Women may select self-employment for reasons that are different from those of men, such as seeking autonomy and flexibility, reducing childcare costs, or reconciling work and family demands (Georgellis and Wall, 2005; Thébaud, 2015). In addition, women may experience well-being outcomes associated with their unique challenges, such as institutional, financial, and networking challenges and barriers. The relationship between women's self-employment and their well-being varies across countries (Crum and Chen, 2015). Entrepreneurial activities are generally associated with degrees of national economic freedom (Lepeley et al., 2019) as well as the labor and employment frameworks in different countries, particularly for women. For example, women's self-employment rate is higher in countries where social welfare systems are less generous, such as in Spain compared with other European countries (Hatfield, 2015), whereas family ties play a key role in how self-employed women pursue their entrepreneurial activities in Asia (Franzke et al., 2022). Despite the different challenges that women entrepreneurs encounter across countries, a general consensus is that the well-being of self-employed women worldwide has a multiplier effect, that is, their well-being is highly correlated with economic empowerment and prosperity, social progress, and sustainability in societies (Lepeley et al., 2019). Well-being research dates back to the 1960s (e.g., Cantril, 1965). However, there is no general consensus as to how empirical studies should measure well-being (Linton et al., 2016). The proposed instruments range from subjective measures of affect and life satisfaction to the measures of objective physical health and social well-being (Wiklund et al., 2019). Among many, the most commonly used measurement of well-being in the self-employment literature is individuals' self-assessment of their life or parts of their life (Abreu et al., 2019). Empirical studies have measured well-being as individuals' satisfaction with their overall life (e.g., Kibler et al., 2019) and/or constituent parts of their life, such as low work-family conflict (Hmieleski and Sheppard, 2019), work satisfaction (Abreu et al., 2019), or satisfaction with health, financial situation, and achievement (Bhuiyan and Ivlevs, 2019). Self-employed individuals' overall well-being tends to be measured as a life evaluation, which is “a reflective rather than a descriptive concept, as it captures individuals' self-assessment of their life based on a standard they choose to be the desirable baseline for themselves” (Abreu et al., 2019, p. 591). Consistent with the above literature and given the available information in our dataset, we adopt a global measure of well-being using individuals' satisfaction with overall life, and further explore well-being in two important constituent parts of life: health and standard of living.^1^

The rest of this paper is organized as follows. Section 2 describes the data and variables and explains the empirical approach. Section 3 presents the main results. Discussion, policy recommendations, and limitations are presented in the last section.

## Data and methods

### Data and variables

Data used in this study come from the Chinese Household Income Project (CHIP) survey conducted in 2014, which collected household and work information from households in 15 provinces in China. The CHIP survey was implemented as part of a collaborative research project on incomes and inequality in China by Chinese and international scholars. We accessed the data through the China Institute for Income Distribution. We restrict our sample to urban households and individuals who work at least 30 hours per week on average, including both wage-employed and self-employed individuals. The sample includes 7,416 (88%) wage-employed and 999 (12%) self-employed individuals.

Well-being is measured using three variables, including self-rated health, satisfaction with standard of living (SOL), and overall life satisfaction, based on three questions in the CHIP survey regarding respondents' satisfaction with their health, SOL, and overall life satisfaction.^2^ The three variables are used as separate outcome variables in our analysis to examine how they are impacted by the type of employment for women and men. As a robustness check, we conducted a confirmatory factor analysis for the three observed variables as indicators of well-being. The results show a good model fit (RMSEA < 0.01, CFI > 0.99, TLI > 0.99, and SRMR < 0.01).

Control variables include age, gender, education level, marital status, the presence of children and elderly in the household, Chinese Communist Party (CCP) membership, Han ethnicity, job tenure, weekly working hours, industry, and geographical regions.

### Methodology

We first conducted a regression analysis within each employment type. Then, we executed an endogenous switching model that applies full-information maximum likelihood (FIML) to simultaneously fit both the selection (self-employment vs. wage-employment) model and the well-being equation model. The regression analysis was conducted using STATA. For the endogenous switching model estimation, we adopted the “movestay” package. This approach helps address the potential endogeneity problem that may exist when variables that affect employment type selection (self-employment vs. wage-employment) also affect employment outcomes (e.g., health or life satisfaction). Following previous literature on entrepreneurship (e.g., Xiu and Gunderson, 2021), we use two instruments to identify selection into types of employment: the father's self-employment in the past and the mother's self-employment in the past. This endogenous switching approach allowed us to calculate a selection-corrected endowment effect and a return effect based on counterfactual measures. The endowment effect shows the well-being difference between wage-employed women and the counterfactual well-being that self-employed women would have if they were wage-employed. In contrast, the return effect captures the change in well-being if self-employed women had chosen wage-employment and shows whether switching from self-employment to wage-employment would have an impact on their well-being.


(1) $$\begin{array}{rcl} \text{Total Effect } & = & \;E\left(Y_{w}|X_{w},\;\beta_{w}\right)\;-\;E\left(Y_{s}|X_{s},\;\beta_{s}\right) \end{array}$$

(2) $$\begin{array}{rcl} \text{Endowment Effect } & = & \;E\left(Y_{w}|X_{w},\;\beta_{w}\right)\;-\;E\left(Y_{s}|X_{s},\;\beta_{w}\right) \end{array}$$

(3) $$\begin{array}{rcl} \text{Returns Effect } & = & \;E\left(Y_{s}|X_{s},\;\beta_{w}\right)\;-\;E\left(Y_{s}|X_{s},\;\beta_{s}\right) \end{array}$$

## Results

### Descriptive statistics

Descriptive statistics and the comparison of well-being by gender and employment type are shown in Table 1. Self-employed individuals reported no higher or lower health than wage-employed individuals when the male and female subsamples were pooled together. However, a further examination by gender showed that self-employed men have better health than wage-employed men, whereas self-employed women have worse health than wage-employed women. Self-employed individuals in general have a higher level of SOL satisfaction, and this difference is larger among men than women. For overall life satisfaction, self-employed men reported higher life satisfaction than wage-employed men, whereas life satisfaction is lower among women in self-employment than in wage-employment.^3^

**Table 1:** Summary statistics and well-being by gender and employment sector.

**Summary statistics of all variables**						
	**Self-employed (*****n*** = **999)**			**Wage-employed (*****n*** = **7,419)**		
	**Mean**		**S.D**.	**Mean**		**S.D**.
Health (max. 5)	4.145		0.774	4.137		0.744
Satisfaction with standard of living (SOL)	3.020		0.779	2.838		0.772
Overall life satisfaction	3.769		0.791	3.771		0.788
Annual earnings (Yuan)	48,974		69,382	39,401		29,151
Female	0.387		0.487	0.420		0.494
Age	43.514		9.093	41.170		9.772
Education (reference = less than middle school)						
Middle school	0.437		0.496	0.224		0.417
High school	0.224		0.417	0.180		0.384
Vocational school	0.091		0.288	0.122		0.327
Two years college	0.102		0.303	0.211		0.408
University and above	0.044		0.205	0.221		0.415
Han ethnicity	0.937		0.243	0.958		0.200
CCP membership	0.069		0.254	0.243		0.429
Job tenure	11.314		8.660	12.995		10.259
Child under 7 in the household	0.177		0.382	0.153		0.360
Child 7–18 in the household	0.432		0.496	0.357		0.479
Senior 66–75 in the household	0.064		0.245	0.060		0.237
Senior above 75 in the household	0.040		0.196	0.040		0.197
Married	0.931		0.254	0.860		0.347
Weekly working hours	57.066		15.362	45.468		8.799
Father self-employed	0.444		0.497	0.283		0.451
Mother self-employed	0.413		0.493	0.283		0.450
**Comparisons of well-being variables by gender and employment sector**						
	**Pooled Sample**					
	**Self-employed**		**Wage-employed**		**Difference**	
	**Mean**	**S.D**.	**Mean**	**S.D**.	**Diff**	***t*** **-value**
Health (max. 5)	4.145	0.774	4.137	0.744	0.009	0.339
SOL satisfaction	3.020	0.779	2.838	0.772	0.182***	6.769
Life satisfaction	3.769	0.791	3.771	0.788	– 0.002	– 0.087
	**Male Subsample**					
	**Self-employed**		**Wage-employed**		**Difference**	
	**Mean**	**S.D**.	**Mean**	**S.D**.	**Diff**	***t*** **-value**
Health (max. 5)	4.196	0.762	4.130	0.755	0.066**	2.011
SOL satisfaction	3.070	0.752	2.828	0.780	0.242***	6.984
Life satisfaction	3.800	0.775	3.762	0.790	0.038	1.100
	**Female Subsample**					
	**Self-Employed**		**Wage-Employed**		**Difference**	
	**Mean**	**S.D**.	**Mean**	**S.D**.	**Diff**	***t*** **-value**
Health (max. 5)	4.065	0.788	4.145	0.728	– 0.081**	– 2.032
SOL satisfaction	2.943	0.815	2.853	0.762	0.090**	2.120
Life satisfaction	3.721	0.814	3.784	0.785	– 0.064	– 1.487

Note: *N* = SOL and Life satisfaction questions are in a separate questionnaire with additional missing values. For SOL, the self-employed *N* = 944 and the wage-employed *N* = 6,877. For life satisfaction, the self-employed *N* = 983 and the wage-employed *N* = 7,271. * < 0.10, ** < 0.05, *** < 0.01.

### Gender differences in well-being within each employment type

Table 2 shows the regression results on well-being without accounting for selection into self-employment. In self-employment, women have lower self-reported health and lower SOL satisfaction than men after controlling for individual and work characteristics. Having higher levels of education, being married, and working fewer hours per week are generally associated with better well-being. In contrast, in wage-employment, women have greater SOL and life satisfaction than men. Being younger, more educated, married, a CCP member, having longer job tenure, and working fewer hours per week are all associated with better well-being.

**Table 2:** Well-being regressions for self-employed and wage-employed.

	**Self-employment**			**Wage-employment**		
	**Health**	**SOL Satisfaction**	**Life Satisfaction**	**Health**	**SOL Satisfaction**	**Life Satisfaction**
Female	– 0.097*	– 0.104*	– 0.039	– 0.012	0.070***	0.055***
	(– 1.898)	(– 1.921)	(– 0.737)	(– 0.638)	(3.544)	(2.836)
Age	0.019	– 0.013	– 0.037	– 0.021***	– 0.033***	– 0.028***
	(0.793)	(– 0.492)	(– 1.458)	(– 2.588)	(– 3.888)	(– 3.325)
Age squared	– 0.000	0.000	0.000	0.000	0.000***	0.000***
	(– 1.538)	(0.319)	(1.127)	(0.597)	(4.044)	(2.726)
Middle school	0.225***	0.119	0.222**	0.109**	0.104**	– 0.028
	(2.607)	(1.300)	(2.455)	(2.368)	(2.047)	(– 0.567)
High school	0.243**	0.202**	0.283***	0.165***	0.156***	0.040
	(2.540)	(1.988)	(2.837)	(3.454)	(2.950)	(0.782)
Vocational school	0.364***	0.066	0.458***	0.151***	0.146***	0.001
	(3.092)	(0.525)	(3.742)	(2.951)	(2.577)	(0.017)
Two years college	0.317***	0.283**	0.093	0.173***	0.233***	0.099*
	(2.761)	(2.324)	(0.778)	(3.486)	(4.243)	(1.839)
University and above	0.359**	0.170	0.348**	0.243***	0.292***	0.161***
	(2.438)	(1.061)	(2.279)	(4.669)	(5.077)	(2.863)
Han ethnicity	0.166	0.015	– 0.024	0.010	0.020	0.035
	(1.524)	(0.126)	(– 0.209)	(0.239)	(0.421)	(0.762)
CCP membership	– 0.017	0.087	0.013	– 0.012	0.081***	0.066***
	(– 0.174)	(0.841)	(0.128)	(– 0.523)	(3.297)	(2.740)
Job tenure	0.010***	0.005	0.007**	0.001	0.004***	0.003***
	(3.150)	(1.425)	(1.989)	(1.047)	(3.669)	(3.069)
Child <7 in household	0.144**	0.029	0.084	0.004	0.050*	0.022
	(2.144)	(0.400)	(1.199)	(0.138)	(1.736)	(0.761)
Child 7–18 in household	0.032	– 0.036	– 0.017	0.046**	0.003	0.003
	(0.578)	(– 0.614)	(– 0.304)	(2.244)	(0.143)	(0.157)
Senior 66–75 in household	0.027	– 0.022	0.089	– 0.005	0.085**	0.042
	(0.265)	(– 0.206)	(0.848)	(– 0.147)	(2.150)	(1.065)
Senior > 75 in household	– 0.004	0.122	0.070	– 0.003	0.013	– 0.043
	(– 0.035)	(0.884)	(0.536)	(– 0.062)	(0.286)	(– 0.920)
Married	– 0.024	0.226**	0.368***	0.105***	0.100***	0.300***
	(– 0.233)	(2.085)	(3.429)	(3.305)	(2.886)	(8.808)
Weekly working hours	– 0.000	– 0.005***	– 0.003*	– 0.002*	– 0.001	– 0.003**
	(– 0.058)	(– 2.786)	(– 1.692)	(– 1.650)	(– 1.046)	(– 2.322)
Industry	Yes	Yes	Yes	Yes	Yes	Yes
Province	Yes	Yes	Yes	Yes	Yes	Yes
Constant	3.744***	3.015***	4.323***	4.500***	2.861***	4.052***
	– 7.406	(5.633)	(8.170)	– 24.769	(14.486)	(20.766)
R^2^	0.139	0.099	0.117	0.070	0.057	0.067
N	999	944	983	7,419	6,877	7,271

Note: * < 0.10, ** < 0.05, *** < 0.01.

We conducted further analyses of the separate male and female subsamples. As shown in Table 3, the results reveal gender differences in the effects of individual and work characteristics on the well-being outcomes. In wage-employment, education has a strong effect on the well-being of both men and women, whereas in self-employment, the effects are generally only statistically significant for men and not women. In self-employment, age is not associated with either men's or women's well-being, but in wage-employment, age has a negative effect on both men's and women's well-being. In self-employment, being married is strongly associated with life satisfaction for men but not for women, whereas in wage-employment, being married is associated with life satisfaction for both men and women, and the coefficient is greater for men than for women. This result echoes research on marriage penalties and premiums and indicates that in addition to wage premiums that men receive from marriage, they also enjoy well-being premiums. As a robustness check, we ran regressions on an identical sample (i.e., observations that responded to all of the three well-being questions). The results are similar to the main findings with different sample sizes for the three outcome variables. We provide the results in the Appendix.

**Table 3:** Well-being regressions for self-employed and wage-employed by gender.

	**Self-employment**						**Wage-employment**					
	**Male**			**Female**			**Male**			**Female**		
	**Health**	**SOL Sat**.	**Life Sat**.	**Health**	**SOL Sat**.	**Life Sat**.	**Health**	**SOL Sat**.	**Life Sat**.	**Health**	**SOL Sat**.	**Life Sat**.
Age	0.022	0.000	– 0.026	0.029	– 0.023	– 0.047	– 0.017	– 0.038***	– 0.016	– 0.036***	– 0.037**	– 0.049***
	(0.732)	(0.004)	(– 0.812)	(0.677)	(– 0.483)	(– 1.025)	(– 1.602)	(– 3.336)	(– 1.389)	(– 2.736)	(– 2.554)	(– 3.435)
Age Squared	<0.001	<0.001	<0.001	– 0.001	<0.001	<0.000	<0.000	<0.000***	<0.000	<0.000*	<0.000***	0.001***
	(– 1.220)	(– 0.132)	(0.577)	(– 1.285)	(0.347)	(0.756)	(– 0.012)	(3.397)	(1.016)	(1.679)	(2.696)	(2.906)
Middle School	0.233**	0.126	0.186	0.202	0.170	0.246*	0.122*	0.113		0.098	0.106	– 0.050
	(1.972)	(1.017)	(1.499)	(1.463)	(1.138)	(1.692)			(– 0.111)	(1.432)	(1.385)	(– 0.670)
High School	0.268**	0.201	0.247*	0.160	0.220	0.320**	0.203***	0.187***	0.049	0.126*	0.114	0.031
	(2.081)	(1.488)	(1.825)	(1.043)	(1.325)	(1.981)	(3.129)	(2.625)	(0.710)	(1.762)	(1.430)	(0.397)
Vocational School	0.448***	0.124	0.563***	0.205	– 0.001	0.313	0.172**	0.158**	0.003	0.132*	0.132	– 0.009
	(2.748)	(0.731)	(3.328)	(1.097)	(– 0.007)	(1.592)	(2.482)	(2.075)	(0.044)	(1.714)	(1.530)	(– 0.105)
2–Years College	0.332**	0.298*	0.125	0.142	0.251	– 0.068	0.200***	0.274***	0.151**	0.147**	0.184**	0.021
	(2.139)	(1.850)	(0.772)	(0.754)	(1.234)	(– 0.345)	(2.969)	(3.703)	(2.085)	(1.965)	(2.214)	(0.257)
Univ. and above	0.501***	0.314*	0.398**	– 0.201	– 0.691*	0.011	0.278***	0.330***	0.207***	0.205***	0.244***	0.082
	(2.858)	(1.702)	(2.185)	(– 0.609)	(– 1.697)	(0.031)	(3.952)	(4.276)	(2.738)	(2.597)	(2.787)	(0.950)
Han ethnicity	0.369**	– 0.010	0.195	– 0.085	0.011	– 0.319*	– 0.014	– 0.040	0.022	0.024	0.089	0.048
	(2.479)	(– 0.069)	(1.266)	(– 0.506)	(0.058)	(– 1.755)	(– 0.237)	(– 0.613)	(0.355)	(0.389)	(1.290)	(0.704)
CCP membership	– 0.059	0.138	– 0.020	0.070	0.031	0.060	0.004	0.066**	0.031	– 0.045	0.107***	0.120***
	(– 0.541)	(1.219)	(– 0.175)	(0.289)	(0.113)	(0.234)	(0.149)	(2.111)	(1.001)	(– 1.200)	(2.662)	(2.987)
Job tenure	0.006	0.004	0.007*	0.018***	0.006	0.006	0.002	0.004***	0.003**	0.001	0.005***	0.004**
	(1.518)	(0.966)	(1.667)	(3.134)	(0.991)	(0.941)	(1.165)	(2.594)	(2.319)	(0.331)	(2.807)	(2.073)
Child <7 in household	0.097	0.022	0.044	0.287**	0.054	0.195	– 0.009	0.038	0.025	0.023	0.062	0.014
	(1.128)	(0.246)	(0.489)	(2.481)	(0.430)	(1.603)	(– 0.257)	(1.023)	(0.696)	(0.544)	(1.361)	(0.314)
Child 7– 18 in household	0.028	– 0.084	– 0.035	0.038	– 0.005	– 0.009	0.058**	– 0.033	– 0.011	0.037	0.052	0.018
	(0.399)	(– 1.139)	(– 0.477)	(0.413)	(– 0.054)	(– 0.090)	(2.086)	(– 1.075)	(– 0.387)	(1.181)	(1.531)	(0.533)
Senior 66–75 in household	0.037	– 0.002	0.067	0.041	– 0.101	0.158	– 0.040	0.102*	0.034	0.042	0.064	0.056
	(0.308)	(– 0.020)	(0.531)	(0.205)	(– 0.473)	(0.750)	(– 0.830)	(1.950)	(0.650)	(0.757)	(1.064)	(0.933)
Senior >75 in household	– 0.047	0.129	0.096	0.073	0.176	0.009	– 0.004	– 0.003	– 0.083	– 0.002	0.037	0.012
	(– 0.307)	(0.792)	(0.603)	(0.310)	(0.623)	(0.038)	(– 0.071)	(– 0.043)	(– 1.369)	(– 0.036)	(0.508)	(0.170)
Married	– 0.019	0.184	0.468***	– 0.119	0.260	0.185	0.095**	0.140***	0.334***	0.125***	0.070	0.268***
	(– 0.132)	(1.272)	(3.164)	(– 0.732)	(1.461)	(1.076)	(2.112)	(2.857)	(6.978)	(2.768)	(1.421)	(5.472)
Weekly working hours	0.002	– 0.006**	– 0.003	– 0.003	– 0.004	– 0.003	– 0.002	– 0.001	– 0.003**	– 0.001	– 0.001	– 0.002
	(0.927)	(– 2.471)	(– 1.334)	(– 1.282)	(– 1.378)	(– 1.083)	(– 1.573)	(– 0.872)	(– 2.382)	(– 0.749)	(– 0.712)	(– 0.886)
Constant	3.281***	2.859***	3.714***	4.261***	3.080***	5.396***	4.565***	2.967***	3.760***	4.557***	2.985***	4.598***
	(5.177)	(4.389)	(5.599)	(4.532)	(2.992)	(5.437)	(18.933)	(11.299)	(14.626)	(15.627)	(9.343)	(14.387)
R^2^	0.152	0.128	0.130	0.179	0.131	0.147	0.084	0.064	0.073	0.064	0.061	0.068
N	612	573.000	600	387	371	383	4,301	3,995	4,213	3,115	2,882	3,058

Note: Industry and Province variables are controlled in all regressions. * < 0.10, ** < 0.05, *** < 0.01.

### Endogenous switching model results

We use the endogenous switching model to account for the selection effect (Lokshin and Sajaia, 2004) and estimate selection-corrected expected well-being. Table 4 shows the switching model results. In Panel A, the estimated total health difference is 0.080, showing that on average, self-employed women have worse health than those who are wage-employed, after controlling for human capital and family characteristics as well as selection into the type of employment. The endowment effect is positive and statistically significant, suggesting that if self-employed women were selected into wage-employment, their self-rated health would not be as good as that of other wage-employed women. The return effect is negative, indicating that self-employed women's own health would be worse if they decided to join wage-employment with the wage-employment health return mechanism applied to them. In essence, there are statistically significant differences in the endowments of health-determining characteristics between self-employed and wage-employed women, and this explains a large portion of the health gap between women in these two employment types. Self-employed women, however, would not achieve health gains if they switched to wage-employment. In contrast, self-employed men have better self-rated health than wage-employed men, after controlling for the effects of other variables and selection into employment type. Different from female entrepreneurs, male entrepreneurs receive large health gains by being self-employed (return effect), which offsets the endowment effect.

**Table 4:** Well-being difference between self-employed and wage-employed: endowments and returns effects.

**Panel A: Health**			
	**Estimated total gap**	**Endowment effect**	**Returns effect**
	*E*(*Y*_*w*_|*X*_*w*_, β_*w*_)− *E*(*Y*_*s*_|*X*_*s*_, β_*s*_)	*E*(*Y*_*w*_|*X*_*w*_, β_*w*_)−*E*(*Y*_*s*_|*X*_*s*_, β_*w*_)	*E*(*Y*_*s*_|*X*_*s*_, β_*w*_)− *E*(*Y*_*s*_|*X*_*s*_, β_*s*_)
Females	0.080***	0.122***	– 0.042***
	(0.010)	(0.009)	(0.014)
Males	– 0.064***	0.169***	– 0.233***
	(0.009)	(0.008)	(0.007)
**Panel B: SOL satisfaction**			
	**Estimated total gap**	**Endowment effect**	**Returns effect**
	*E*(*Y*_*w*_|*X*_*w*_, β_*w*_)− *E*(*Y*_*s*_|*X*_*s*_, β_*s*_)	*E*(*Y*_*w*_|*X*_*w*_, β_*w*_)−*E*(*Y*_*s*_|*X*_*s*_, β_*w*_)	*E*(*Y*_*s*_|*X*_*s*_, β_*w*_)− *E*(*Y*_*s*_|*X*_*s*_, β_*s*_)
Females	– 0.086***	0.226***	– 0.313***
	(0.009)	(0.009)	(0.010)
Males	– 0.244***	0.254***	– 0.497***
	(0.007)	(0.007)	(0.008)
**Panel C: Life satisfaction**			
	**Estimated total gap**	**Endowment effect**	**Returns effect**
	*E*(*Y*_*w*_|*X*_*w*_, β_*w*_)− *E*(*Y*_*s*_|*X*_*s*_, β_*s*_)	*E*(*Y*_*w*_|*X*_*w*_, β_*w*_)−*E*(*Y*_*s*_|*X*_*s*_, β_*w*_)	*E*(*Y*_*s*_|*X*_*s*_, β_*w*_)− *E*(*Y*_*s*_|*X*_*s*_, β_*s*_)
Females	0.065***	0.093***	– 0.028***
	(0.010)	(0.009)	(0.010)
Males	– 0.037***	0.204***	– 0.242***
	(0.008)	(0.008)	(0.007)

Note: * < 0.10, ** < 0.05, *** < 0.01.

The endogenous switching model results for SOL and overall life satisfaction are presented in Panels B and C, respectively. Controlling for human capital and work characteristics as well as selection into the type of employment, SOL satisfaction is higher among self-employed women than wage-employed women, and this pattern is similar for men. In contrast, life satisfaction is greater among wage-employed women than self-employed women, whereas it is lower among wage-employed men than self-employed men.

In summary, the endowment and return effects for women across the three well-being measures are generally consistent; that is, after controlling for selection into wage-employment and self-employment, self-employed women have lower levels of well-being compared with self-employed men, and they also have lower levels of health and life satisfaction than wage-employed women.

## Discussion

Our study finds that self-employed women have lower well-being (health, SOL, and life satisfaction) compared with self-employed men, and they also have lower levels of health and life satisfaction than wage-employed women, after controlling for selection into employment types. In contrast, self-employed men have a better well-being than wage-employed men. Switching to wage-employment would not help enhance self-employed women's well-being.

These findings contribute to the existing literature in two ways. First, our study shows the heterogeneity of well-being outcomes of self-employment for women and men, which provides validation of previous studies that generally show a positive correlation between self-employment and well-being without examining differences between genders (e.g., Abreu et al., 2019). Second, this study expands the scope of the gender pay literature by considering well-being outcomes. Studies show that women in China receive less pay than men in different employment types and occupations across the pay distribution (e.g., Xiu and Gunderson, 2021). Our findings extend this literature by showing that women not only suffer worse economic outcomes than men but also fare poorly on well-being outcomes in employment.

Our research has several policy implications. First, policies should be developed to promote gender equality in both self-employment and wage-employment. The finding that women experience lower levels of well-being than men in both types of employment indicates that policies, such as childcare support and maternity leave, efforts to reduce or eliminate gender discrimination, and promotion of equity-based gender attitudes in both wage-employment and self-employment (e.g., networking, fund-raising, hiring, promotion, and compensation) are imperative (Cebi and Wang, 2013; Jin et al., 2016; Cooke, 2022). These policies will stimulate positive outcomes beyond the economy and help foster a level playing field for all. Second, policies promoting self-employment as a pathway in achieving better well-being outcomes should be coupled with more support for self-employed women to capture the multiplier effect of well-being of self-employed women (Lepeley et al., 2019). Women would benefit from programs designed to inform them about opportunities and challenges associated with self-employment so they can make more informed career decisions. In addition, policies designed to help self-employed women find the balance between family and work could increase their well-being.

Several limitations of our study should be noted. First, the study restricts the sample to individuals who work more than 30 h/week. Thus, our findings may not generalize to workers and self-employed individuals who work part-time. Future studies applying our methods to part-time employment would be useful. Second, the data used in this study were collected in 2014. We chose this dataset because this national survey contains variables that are rarely included in other national surveys, such as each parent's self-employment status, which serves as an instrumental variable to identify selection into employment type. Therefore, our findings do not reflect the impact of recent labor market changes (e.g., COVID-19). One promising direction for future research would be to address how the pandemic affects women's and men's well-being outcomes in self-employment and wage-employment. Third, our robustness check on non-responses for the three outcome variables shows that although gender and employment type did not impact whether participants responded to overall life satisfaction and health questions, wage-employed individuals were slightly less likely to respond to the SOL satisfaction question. Our results for SOL satisfaction, therefore, could be biased due to the missing data pattern for this variable. Future studies on well-being may explore whether certain groups of individuals are more or less likely to respond to questions on different aspects of well-being. Furthermore, although the current study shows negative well-being outcomes associated with self-employment for women, this effect may vary across contingent factors, such as time, location, and age. Accordingly, future research is encouraged to develop a comprehensive model that addresses the influence of self-employment on the well-being of women and men with consideration of these contingent factors.

## Data availability statement

Publicly available datasets were analyzed in this study. This data can be found here: http://www.ciidbnu.org/chip/index.asp?lang=EN.

## Author contributions

All authors listed have made a substantial, direct, and intellectual contribution to the work and approved it for publication.

## Conflict of interest

The authors declare that the research was conducted in the absence of any commercial or financial relationships that could be construed as a potential conflict of interest.

## Publisher's note

All claims expressed in this article are solely those of the authors and do not necessarily represent those of their affiliated organizations, or those of the publisher, the editors and the reviewers. Any product that may be evaluated in this article, or claim that may be made by its manufacturer, is not guaranteed or endorsed by the publisher.

## Footnotes

^1^In a recent review article, Linton et al. (2016) identified 99 measurement instruments for well-being. We acknowledge that similar to most studies in the literature, our measures of well-being do not encompass all aspects of well-being; instead, we use an overall well-being measure (i.e., life satisfaction) and additionally address health and financial aspects of well-being.

^2^Single-item satisfaction measures, such as life satisfaction, are consistent with the general approach used in the well-being literature, that is, the concept of satisfaction with the components of well-being relevant to one's own global life evaluation, rather than a criterion that researchers may deem important (Kibler et al., 2019). Additionally, previous studies show that single-item satisfaction measures (e.g., life satisfaction) tend to have similar validity to multiple-item measures (e.g., Cheung and Lucas, 2014). Specifically, the self-rated health variable is based on a question that asks respondents about their health compared with an average person in their age group, using a five-point scale ranging from “very poor” to “very good.” The SOL satisfaction variable is based on the question “how do you rate your standard of living compared with others?” using a five-point scale ranging from “much lower” to “much higher.” Overall life satisfaction is based on the question “considering all aspects of life, are you happy?” using a five-point scale ranging from “not happy at all” to “very happy”.

^3^We check for non-response bias by creating dummy variables for non-responses for the three outcome variables and run logistic regressions to check whether the pattern of non-responses is related to gender and self-employment. Gender has no statistically significant relationship with non-response for all three outcome variables. Self-employment has no significant relationship with non-response to the questions on overall life satisfaction and health. The only statistically significant finding is that wage-employed individuals are slightly less likely to respond to the question on SOL satisfaction (correlation = 0.022, *p* = 0.041).
