# Supplementary material for: Gain or loss? The well-being of women in self-employment
Source: Front Psychol. 2022 Sep 16;13:986288. doi: 10.3389/fpsyg.2022.986288 (PMC9523742; doi:10.3389/fpsyg.2022.986288)
Supplement: Supplementary file 1 [file Data_Sheet_1.docx]

**Appendix Table 1. Well-Being Regressions for Self-Employed and Wage-Employed (on the same sample size)**

|  |  | Self-Employment | | | |  | | Wage-Employment | | | |
| --- | --- | --- | --- | --- | --- | --- | --- | --- | --- | --- | --- |
|  |  | Health | SOL Satisfaction | Life Satisfaction | | | Health | | SOL Satisfaction | Life Satisfaction |  |
| Female |  | -0.102* | -0.104* | -0.050 |  | | -0.016 | | 0.068*** | 0.060*** |  |
|  |  | (-1.960) | (-1.921) | (-0.911) |  | | (-0.865) | | (3.440) | (3.017) |  |
| Age |  | 0.018 | -0.013 | -0.040 |  | | -0.017** | | -0.033*** | -0.029*** |  |
|  |  | (0.740) | (-0.492) | (-1.547) |  | | (-2.059) | | (-3.776) | (-3.358) |  |
| Age Squared |  | -0.000 | 0.000 | 0.000 |  | | 0.000 | | 0.000*** | 0.000*** |  |
|  |  | (-1.431) | (0.319) | (1.253) |  | | (0.144) | | (3.916) | (2.872) |  |
| Middle School |  | 0.203** | 0.119 | 0.274*** |  | | 0.119** | | 0.099* | -0.038 |  |
|  |  | (2.296) | (1.300) | (2.959) |  | | (2.453) | | (1.935) | (-0.741) |  |
| High School |  | 0.231** | 0.202** | 0.330*** |  | | 0.179*** | | 0.150*** | 0.036 |  |
|  |  | (2.370) | (1.988) | (3.225) |  | | (3.549) | | (2.835) | (0.667) |  |
| Vocational School | | 0.363*** | 0.066 | 0.519*** |  | | 0.157*** | | 0.136** | -0.006 |  |
|  |  | (3.018) | (0.525) | (4.124) |  | | (2.911) | | (2.392) | (-0.099) |  |
| 2-years College | | 0.312*** | 0.283** | 0.110 |  | | 0.176*** | | 0.229*** | 0.111** |  |
|  |  | (2.669) | (2.324) | (0.898) |  | | (3.374) | | (4.173) | (1.994) |  |
| Univ. & Above |  | 0.363** | 0.170 | 0.400** |  | | 0.258*** | | 0.282*** | 0.168*** |  |
|  |  | (2.360) | (1.061) | (2.482) |  | | (4.709) | | (4.904) | (2.864) |  |
| Han ethnicity |  | 0.141 | 0.015 | 0.033 |  | | 0.024 | | 0.022 | 0.054 |  |
|  |  | (1.260) | (0.126) | (0.280) |  | | (0.529) | | (0.472) | (1.127) |  |
| CPP Membership | | -0.030 | 0.087 | 0.032 |  | | -0.015 | | 0.081*** | 0.058** |  |
|  |  | (-0.306) | (0.841) | (0.312) |  | | (-0.643) | | (3.295) | (2.312) |  |
| Job Tenure |  | 0.010*** | 0.005 | 0.007** |  | | 0.002 | | 0.004*** | 0.003*** |  |
|  |  | (3.157) | (1.425) | (2.052) |  | | (1.398) | | (3.800) | (2.867) |  |
| Child < 7 in Household | | 0.143** | 0.029 | 0.095 |  | | 0.006 | | 0.048* | 0.021 |  |
|  |  | (2.071) | (0.400) | (1.318) |  | | (0.214) | | (1.684) | (0.721) |  |
| Child 7-18 in Household | | 0.032 | -0.036 | -0.016 |  | | 0.043** | | -0.002 | 0.006 |  |
|  |  | (0.567) | (-0.614) | (-0.279) |  | | (2.038) | | (-0.074) | (0.253) |  |
| Senior 66-75 in Household | | 0.014 | -0.022 | 0.134 |  | | -0.015 | | 0.083** | 0.036 |  |
|  |  | (0.140) | (-0.206) | (1.242) |  | | (-0.390) | | (2.104) | (0.901) |  |
| Senior > 75 in Household | | 0.077 | 0.122 | 0.040 |  | | 0.012 | | 0.015 | -0.055 |  |
|  |  | (0.581) | (0.884) | (0.286) |  | | (0.263) | | (0.312) | (-1.157) |  |
| Married |  | -0.002 | 0.226** | 0.407*** |  | | 0.093*** | | 0.098*** | 0.293*** |  |
|  |  | (-0.017) | (2.085) | (3.724) |  | | (2.834) | | (2.827) | (8.341) |  |
| Weekly working hours | | -0.000 | -0.005*** | -0.003* |  | | -0.001 | | -0.001 | -0.002* |  |
|  |  | (-0.303) | (-2.786) | (-1.709) |  | | (-0.956) | | (-0.951) | (-1.726) |  |
| Industry |  | Yes | Yes | Yes |  | | Yes | | Yes | Yes |  |
| Provinces |  | Yes | Yes | Yes |  | | Yes | | Yes | Yes |  |
| constant |  | 3.754*** | 3.015*** | 4.270*** |  | | 4.342*** | | 2.846*** | 4.013*** |  |
|  |  | (7.291) | (5.633) | (7.922) |  | | (23.094) | | (14.420) | (19.976) |  |
| R^2^ |  | 0.140 | 0.099 | 0.130 |  | | 0.074 | | 0.058 | 0.068 |  |
| N |  | 944 | 944 | 944 |  | | 6854 | | 6854 | 6854 |  |

Note: *<0.10, **<0.05, ***<0.01

**Appendix Table 2. Well-Being Regressions for Self-Employed and Wage-Employed by Gender (on the same sample size)**

|  |  | Self-Employment | | | | | | | | | | |  | | Wage-Employment | | | | | | | | | | | | | | |
| --- | --- | --- | --- | --- | --- | --- | --- | --- | --- | --- | --- | --- | --- | --- | --- | --- | --- | --- | --- | --- | --- | --- | --- | --- | --- | --- | --- | --- | --- |
|  |  | Male | | | |  | | Female | | | | |  | | Male | | | | |  | | Female | | | | |  |  |  |
|  |  | Health | SOL Sat. | Life Sat. | | | Health | | SOL Sat. | | Life Sat. | | | Health | | SOL Sat. | | Life Sat. | | | Health | | SOL Sat. | | Life Sat. | | |  |  |
| Age |  | 0.012 | 0.000 | -0.031 |  | | 0.042 | | | -0.023 | | -0.048 |  | | -0.012 | | -0.037*** | | -0.017 |  | | -0.034** | | -0.036** | | -0.053*** | | |  |
|  |  | (0.384) | (0.004) | (-0.973) |  | | (0.946) | | | (-0.483) | | (-1.020) |  | | (-1.122) | | (-3.216) | | (-1.429) |  | | (-2.463) | | (-2.523) | | (-3.566) | | |  |
| Age Squared | | -0.000 | -0.000 | 0.000 |  | | -0.001 | | | 0.000 | | 0.000 |  | | -0.000 | | 0.000*** | | 0.000 |  | | 0.000 | | 0.000*** | | 0.001*** | | |  |
|  |  | (-0.780) | (-0.132) | (0.763) |  | | (-1.572) | | | (0.347) | | (0.777) |  | | (-0.409) | | (3.263) | | (1.125) |  | | (1.438) | | (2.657) | | (3.137) | | |  |
| Middle School | | 0.207* | 0.126 | 0.269** |  | | 0.176 | | | 0.170 | | 0.262* |  | | 0.129* | | 0.104 | | -0.018 |  | | 0.112 | | 0.105 | | -0.053 | | |  |
|  |  | (1.696) | (1.017) | (2.116) |  | | (1.268) | | | (1.138) | | (1.774) |  | | (1.953) | | (1.499) | | (-0.261) |  | | (1.536) | | (1.372) | | (-0.667) | | |  |
| High School | | 0.254* | 0.201 | 0.321** |  | | 0.145 | | | 0.220 | | 0.341** |  | | 0.211*** | | 0.179** | | 0.046 |  | | 0.147* | | 0.112 | | 0.035 | | |  |
|  |  | (1.911) | (1.488) | (2.312) |  | | (0.936) | | | (1.325) | | (2.073) |  | | (3.101) | | (2.495) | | (0.633) |  | | (1.927) | | (1.402) | | (0.427) | | |  |
| Vocational School | | 0.477*** | 0.124 | 0.678*** |  | | 0.166 | | | -0.001 | | 0.309 |  | | 0.180** | | 0.143* | | -0.017 |  | | 0.136* | | 0.128 | | 0.015 | | |  |
|  |  | (2.863) | (0.731) | (3.896) |  | | (0.878) | | | (-0.007) | | (1.532) |  | | (2.464) | | (1.865) | | (-0.215) |  | | (1.663) | | (1.489) | | (0.169) | | |  |
| 2-years College | | 0.350** | 0.298* | 0.189 |  | | 0.130 | | | 0.251 | | -0.071 |  | | 0.207*** | | 0.267*** | | 0.162** |  | | 0.146* | | 0.184** | | 0.047 | | |  |
|  |  | (2.203) | (1.850) | (1.139) |  | | (0.686) | | | (1.234) | | (-0.354) |  | | (2.928) | | (3.605) | | (2.170) |  | | (1.837) | | (2.208) | | (0.545) | | |  |
| Univ. & Above | | 0.509*** | 0.314* | 0.505*** |  | | -0.361 | | | -0.691* | | -0.156 |  | | 0.284*** | | 0.316*** | | 0.212*** |  | | 0.230*** | | 0.240*** | | 0.105 | | |  |
|  |  | (2.800) | (1.702) | (2.655) |  | | (-0.951) | | | (-1.697) | | (-0.387) |  | | (3.854) | | (4.079) | | (2.706) |  | | (2.751) | | (2.741) | | (1.160) | | |  |
| Han Ethnicity | | 0.351** | -0.010 | 0.257 |  | | -0.155 | | | 0.011 | | -0.313* |  | | 0.006 | | -0.037 | | 0.049 |  | | 0.032 | | 0.090 | | 0.061 | | |  |
|  |  | (2.346) | (-0.069) | (1.643) |  | | (-0.872) | | | (0.058) | | (-1.651) |  | | (0.103) | | (-0.570) | | (0.745) |  | | (0.479) | | (1.314) | | (0.862) | | |  |
| CCP Membership | | -0.087 | 0.138 | -0.002 |  | | 0.117 | | | 0.031 | | 0.127 |  | | 0.000 | | 0.066** | | 0.018 |  | | -0.044 | | 0.106*** | | 0.116*** | | |  |
|  |  | (-0.783) | (1.219) | (-0.017) |  | | (0.454) | | | (0.113) | | (0.464) |  | | (0.007) | | (2.120) | | (0.565) |  | | (-1.157) | | (2.648) | | (2.819) | | |  |
| Job Tenure | | 0.007 | 0.004 | 0.007* |  | | 0.017*** | | | 0.006 | | 0.006 |  | | 0.002 | | 0.004*** | | 0.003** |  | | 0.001 | | 0.006*** | | 0.004** | | |  |
|  |  | (1.645) | (0.966) | (1.717) |  | | (2.976) | | | (0.991) | | (1.065) |  | | (1.357) | | (2.681) | | (2.031) |  | | (0.593) | | (2.904) | | (2.098) | | |  |
| Child < 7 in Household | | 0.116 | 0.022 | 0.041 |  | | 0.263** | | | 0.054 | | 0.238* |  | | -0.002 | | 0.034 | | 0.024 |  | | 0.019 | | 0.064 | | 0.016 | | |  |
|  |  | (1.299) | (0.246) | (0.444) |  | | (2.244) | | | (0.430) | | (1.913) |  | | (-0.051) | | (0.920) | | (0.628) |  | | (0.435) | | (1.410) | | (0.345) | | |  |
| Child 7-18 in Household | | 0.029 | -0.084 | -0.041 |  | | 0.028 | | | -0.005 | | -0.010 |  | | 0.059** | | -0.038 | | -0.011 |  | | 0.032 | | 0.048 | | 0.023 | | |  |
|  |  | (0.393) | (-1.139) | (-0.536) |  | | (0.300) | | | (-0.054) | | (-0.099) |  | | (2.037) | | (-1.256) | | (-0.353) |  | | (0.986) | | (1.422) | | (0.651) | | |  |
| Senior 66-75 in Household | | 0.039 | -0.002 | 0.119 |  | | 0.003 | | | -0.101 | | 0.178 |  | | -0.041 | | 0.100* | | 0.029 |  | | 0.025 | | 0.064 | | 0.050 | | |  |
|  |  | (0.317) | (-0.020) | (0.923) |  | | (0.016) | | | (-0.473) | | (0.839) |  | | (-0.833) | | (1.906) | | (0.550) |  | | (0.427) | | (1.050) | | (0.803) | | |  |
| Senior > 75 in Household | | 0.043 | 0.129 | 0.075 |  | | 0.181 | | | 0.176 | | -0.070 |  | | 0.021 | | 0.002 | | -0.094 |  | | -0.003 | | 0.034 | | 0.000 | | |  |
|  |  | (0.272) | (0.792) | (0.451) |  | | (0.688) | | | (0.623) | | (-0.251) |  | | (0.355) | | (0.038) | | (-1.505) |  | | (-0.039) | | (0.469) | | (0.001) | | |  |
| Married |  | 0.024 | 0.184 | 0.498*** |  | | -0.106 | | | 0.260 | | 0.259 |  | | 0.066 | | 0.135*** | | 0.324*** |  | | 0.131*** | | 0.071 | | 0.267*** | | |  |
|  |  | (0.171) | (1.272) | (3.336) |  | | (-0.636) | | | (1.461) | | (1.471) |  | | (1.422) | | (2.753) | | (6.556) |  | | (2.798) | | (1.451) | | (5.287) | | |  |
| Weekly working hours | | 0.002 | -0.006** | -0.003 |  | | -0.004 | | | -0.004 | | -0.003 |  | | -0.001 | | -0.001 | | -0.003** |  | | -0.000 | | -0.001 | | -0.000 | | |  |
|  |  | (0.786) | (-2.471) | (-1.402) |  | | (-1.451) | | | (-1.378) | | (-1.042) |  | | (-1.075) | | (-0.728) | | (-2.237) |  | | (-0.275) | | (-0.763) | | (-0.138) | | |  |
| constant |  | 3.383*** | 2.859*** | 3.715*** |  | | 4.144*** | | | 3.080*** | | 5.271*** |  | | 4.385*** | | 2.946*** | | 3.733*** |  | | 4.449*** | | 2.980*** | | 4.567*** | | |  |
|  |  | (5.271) | (4.389) | (5.540) |  | | (4.318) | | | (2.992) | | (5.169) |  | | (17.533) | | (11.223) | | (14.064) |  | | (14.592) | | (9.332) | | (13.906) | | |  |
| R^2^ |  | 0.149 | 0.128 | 0.146 |  | | 0.192 | | | 0.131 | | 0.161 |  | | 0.086 | | 0.065 | | 0.075 |  | | 0.067 | | 0.061 | | 0.069 | | |  |
| N |  | 573 | 573 | 573 |  | | 371 | | | 371 | | 371 |  | | 3981 | | 3981 | | 3981 |  | | 2873 | | 2873 | | 2873 | | |  |

Note: Industry and province variables are controlled in all regressions. *<0.10, **<0.05, ***<0.01
